# The Impact of Spatial Structure on Viral Genomic Diversity Generated during Adaptation to Thermal Stress

**DOI:** 10.1371/journal.pone.0088702

**Published:** 2014-02-12

**Authors:** Dilara Ally, Valorie R. Wiss, Gail E. Deckert, Danielle Green, Pavitra Roychoudhury, Holly A. Wichman, Celeste J. Brown, Stephen M. Krone

**Affiliations:** 1 Department of Biological Sciences, University of Idaho, Moscow, Idaho, United States of America; 2 Institute for Bioinformatics and Evolutionary Studies, University of Idaho, Moscow, Idaho, United States of America; 3 Department of Veterinary Microbiology and Pathology, Washington State University, Pullman, Washington, United States of America; 4 Department of Mathematics, University of Idaho, Moscow, Idaho, United States of America; Leiden University, Netherlands

## Abstract

**Background:**

Most clinical and natural microbial communities live and evolve in spatially structured environments. When changes in environmental conditions trigger evolutionary responses, spatial structure can impact the types of adaptive response and the extent to which they spread. In particular, localized competition in a spatial landscape can lead to the emergence of a larger number of different adaptive trajectories than would be found in well-mixed populations. Our goal was to determine how two levels of spatial structure affect genomic diversity in a population and how this diversity is manifested spatially.

**Methodology/Principal Findings:**

We serially transferred bacteriophage populations growing at high temperatures (40°C) on agar plates for 550 generations at two levels of spatial structure. The level of spatial structure was determined by whether the physical locations of the phage subsamples were preserved or disrupted at each passage to fresh bacterial host populations. When spatial structure of the phage populations was preserved, there was significantly greater diversity on a global scale with restricted and patchy distribution. When spatial structure was disrupted with passaging to fresh hosts, beneficial mutants were spread across the entire plate. This resulted in reduced diversity, possibly due to clonal interference as the most fit mutants entered into competition on a global scale. Almost all substitutions present at the end of the adaptation in the populations with disrupted spatial structure were also present in the populations with structure preserved.

**Conclusions/Significance:**

Our results are consistent with the patchy nature of the spread of adaptive mutants in a spatial landscape. Spatial structure enhances diversity and slows fixation of beneficial mutants. This added diversity could be beneficial in fluctuating environments. We also connect observed substitutions and their effects on fitness to aspects of phage biology, and we provide evidence that some substitutions exclude each other.

## Introduction

Microbial communities typically live and evolve in spatial environments. Spatial structure, which can arise as a consequence of limited dispersal, environmental variation [1] or because of spatially explicit interactions [2], promotes heterogeneity in populations. A key feature of spatial structure is the localization of the interactions between organisms. In particular, individuals within spatially structured environments are not subject to global competition [3].

Spatial structure effectively creates multiple, pseudo-independent subpopulations. This means that different mutations may arise and have the potential to fix in different regions of the spatial landscape, leading to parallel adaptation especially in situations with a global selective pressure [4]. Because subpopulations are smaller, large-effect beneficial mutations that dominate in well-mixed populations [5]–[9] could play a reduced role relative to genetic drift [10]–[12]. Thus, spatial structure may promote a richer palette of adaptive trajectories from which the population may draw. This could be especially important in situations where environmental conditions fluctuate. Although there has been considerable effort aimed at exploring the effects of spatial structure on evolutionary dynamics [4], [10], [11], [13], [14], empirical studies are relatively rare [12], [15], [16], especially at the genomic level.

As an example of how spatial structure can affect evolutionary dynamics, consider the case of clonal interference. Well-mixed populations, in which dispersal is widespread and individuals freely interact, are characterized by global competition. With the extremely large population sizes and high mutation rates that are typical of microbial populations, global competition can give rise to clonal interference [17] in which a mutant that begins a selective sweep can be outcompeted by a later-arising and more-fit mutant before the original sweep is complete [9]. These competition dynamics can favour larger-effect beneficial mutations, as well as decreasing genetic variability within a population.

Clonal interference was originally characterized by Gerrish and Lenski [17] as a concept pertaining to well-mixed populations. Under assumptions commonly used to analyze clonal interference, involving the relative rates at which new beneficial mutants arise and their fitness benefits, one does not distinguish between the failure of a mutant to sweep to fixation and its being driven to extinction through the fixation of a more-fit mutant. In spatially distributed populations, these notions are not the same. If one interprets clonal interference in a spatial context as the failure of beneficial mutants to sweep to fixation, as opposed to the loss of these mutants, then spatial structure should increase clonal interference [18], [19]. Kryazhimskiy *et al*. [18] refer to the trade-off between “exploration” (of more evolutionary possibilities when competition is muted due to structure) and “exploitation” (in which fixing certain mutations might be preferable) and how these aspects of adaptation can be mediated by the degree of population subdivision.

While some theoretical and empirical work has emphasized the impact of spatial structure on phenotypic diversity, most of that work either examines phenotypic characters correlated to fitness or how spatial structure affects the fixation rate of a given beneficial mutant [3], [10]–[12], [15], [20]–[24]. Moreover, theory is typically built on simplifying assumptions about the fitness effects of mutations; these assumptions are usually at most tangentially grounded in the biology. For example, Gordo and Campos [10] and Habets et al. [11] use simple spatial models to predict that increased spatial structure leads to a slowing down of the fixation rate of beneficial mutants in bacterial populations. These models either address the fate of a single beneficial mutant or assume that fitness effects of multiple mutations are independent of one another and exponentially distributed. The model of Habets et al. [11] also predicts that the initial distribution (localized or mixed) also plays a role in the probability of fixation of a beneficial mutant. Habets et al. [11] and Perfeito et al. [12] demonstrated the predicted reduction in the rate of adaptation with increased spatial structure in experimental *E. coli* populations. These authors also suggest that clonal interference should play a significant role in spatial adaptation due to the large population sizes and high mutation rates in microbial populations, but this was less developed and the distinction between the different interpretations in the above paragraph was not addressed. Ralph and Coop [4] carried out another theoretical study of relevance to the present paper. They used a continuous spatial model to argue that parallel adaptation (via different mutations of similar phenotypic effect) would be enhanced by spatial structure.

Here we seek to determine how spatial structure will affect the magnitude and pattern of *adaptive genetic variation* in a virus that is adapting to a change in environment. Our experimental model system consists of the bacteriophage ID11 and its bacterial host, *E. coli*, and the environmental shift is an increase in temperature. Additionally, since ID11 was originally captured in environmental samples from an unknown host, adaptation to *E. coli* in a laboratory environment may also be occurring. ID11 grows optimally at 33°C; we chose 40°C as the selective temperature because this was identified as the limit of the thermal niche for ID11 [25].

Because our intent was to examine how spatial structure influences genetic diversity during adaptive evolution, we used two levels of spatial structure: “structured-transfer” and “mixed-transfer.” In our design, spatial structure was realized by growing viral populations with *E. coli* hosts in semi-solid agar on the surface of an agar plate. Treatment differences were based on the method of serial transfer of viral subpopulations employed after each incubation period. In the structured-transfer treatments, a replicate picker was used to maintain the relative locations of the sampled phage subpopulations when they were transferred to a lawn of fresh host cells. In mixed-transfer treatments, spatial structure was disrupted prior to transfer using the replicate picker.

## Results

**Figure 1:**
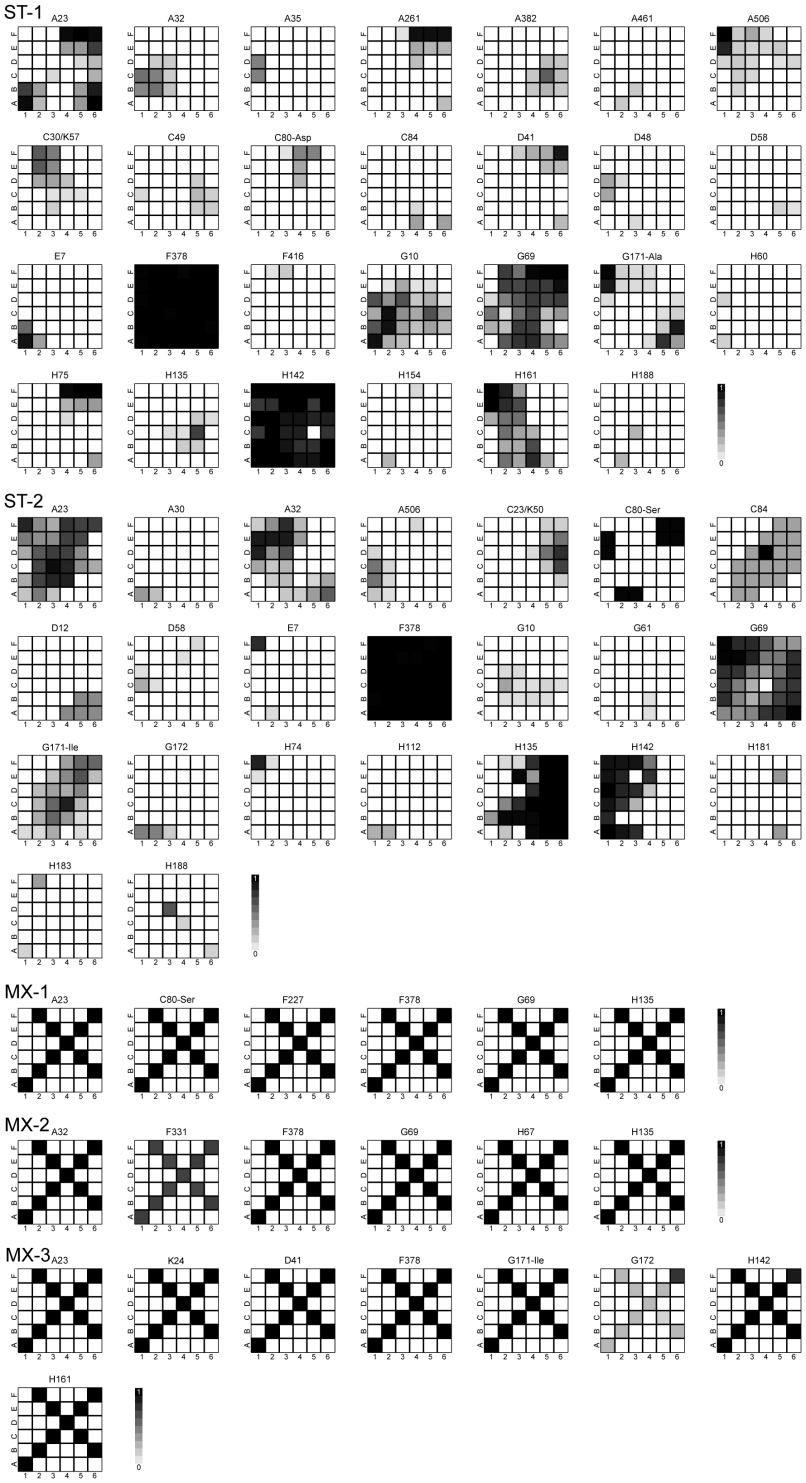
Spatial distribution of mutations after 550 generations of adaptation to high temperature (40°C). The spatial arrangement of all substitutions in each of two replicates for structured-transfer plates (ST-1, ST-2) and for three mixed-transfer plates (MX-1, MX-2, MX-3) are shown. Presented above each grid is the location in the protein of each substitution and, when necessary, the amino acid substitution. Silent substitutions are in italics. Squares within a grid are shaded in increments of 10% based upon the fraction of 454 reads with the substitution; white denotes absence and black fixation. Preliminary sequencing of mixed-transfer subpopulations indicated that most substitutions were spread across the plate, so we only tracked substitutions for MX replicates in the squares indicated by the X.

To analyze the genetic diversity in our evolution experiments, we sequenced subpopulations from all three replicates in both the structured-transfer (ST) and mixed-transfer (MX) plates at the final time point of the experiment (generation 550). The different replicates were labeled ST-1, ST-2, ST-3 for structured transfer, and MX-1, MX-2, MX-3 for mixed transfer. During the post-experiment analysis of the data, there was evidence that ST-3 might have been switched with a replicate of a parallel experiment (not treated here). Data were analyzed both with and without the ST-3 replicate; the statistical conclusions are the same although conclusions about the identities of mutations are, of course, affected. To be conservative, ST-3 was removed from the analysis. Populations from individual squares on a plate were sequenced to obtain spatial resolution. Although each square had at least 50x coverage, 454 sequencing errors are unavoidable. To minimize the impact of sequencing errors on our conclusions, we did not include singletons (substitutions that were detected in only one square per plate) in any of the analyses. This provides a conservative estimate of the effect of spatial structure on genetic variation in these experiments. For the ST populations, we sequenced from each of the 36 squares. For the MX populations, preliminary Sanger sequencing showed that each substitution that rose to a detectable frequency was actually found in all the squares. This is not surprising since mixed-transfer passaging resulted in mutants being spread across the entire plate after each of the 50 incubation periods. For this reason, we sequenced subsamples from only 10 of the squares in each of the MX plates (indicated by the “X” pattern in the MX plates of Figure 1). As the results in Figure 1 show, there was no variation between the sampled squares on any of the MX plates, consistent with the preliminary sequencing showing global spread of all MX substitutions.

### Higher Global Genetic Diversity in Structured-Transfer than in Mixed-Transfer Treatments

**Figure 2:**
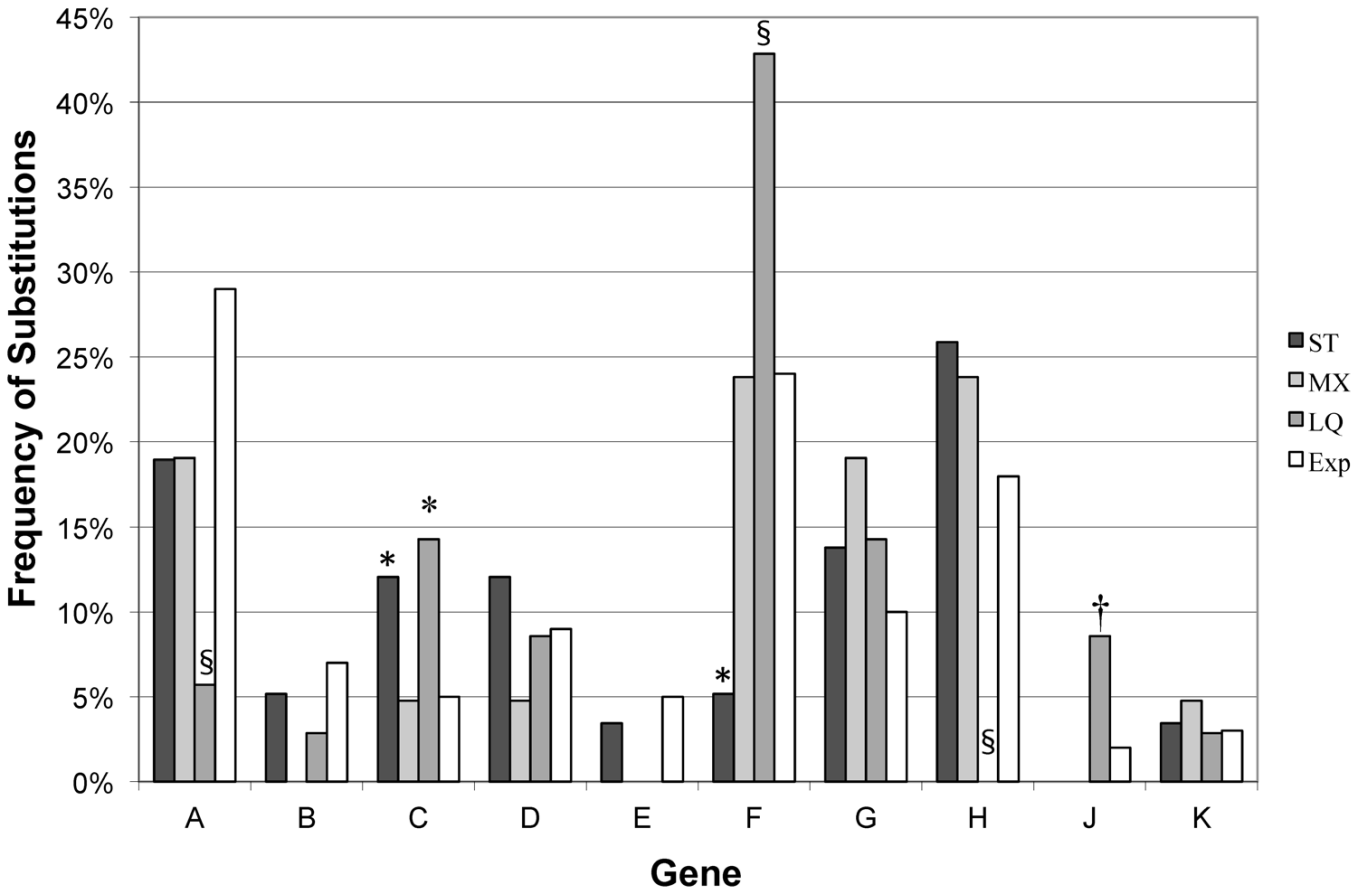
Locations of mutations across the genome of ID11. Comparison of the percentage of mutations found in each gene for both spatial treatments (ST and MX) evolved for 550 generations at 40°C, and a 37°C liquid evolution experiment (LQ) after 40–60 generations [27]. These percentages are also compared to the percentage of the genome encoded by the different genes (Exp). MX experiments did not show a significant departure from expectation based upon the length of each gene. ST experiments had a greater number of substitutions in C and fewer in A than expected by chance. Liquid experiments found a disproportionate number of mutations in C, F and J, and fewer than would be expected by chance in genes A and H. Symbols indicate significant p-values for a chi-square test (1 df) of departures from expectation for each gene. § p-value <0.025, * p-value <0.01, † p-value <0.005.

Across all treatments and replicates, we observed a total of 43 unique nucleotide mutations that translated to 30 different amino acid substitutions at the final time point (Table 1, Figure 1). These substitutions were found in more than one square per plate, and hence are unlikely to be attributable to sequencing error. Across the two replicates of our structured-transfer treatments, we found a total of 39 unique nucleotide substitutions (26 amino acid changes). In contrast, only 15 unique nucleotide substitutions (14 amino acid changes) were found across the three replicates of the mixed-transfer treatment (Table 1). Thus, there was about three times more global diversity in the structured-transfer experiments. The total number of substitutions, counting occurrences from each replicate in which they occur, was 71, with 51 of these arising in the ST populations and 20 arising in the MX populations (Table 1, last row). Some of these substitutions appeared to exclude each other (Table 2) and there was a fairly broad scattering of mutations across the genome (Figure 2), as will be discussed below.

**Table 1:** Sequence variants from structured and mixed-transfer plates evolved for 550 generations.

			Ancestor		Evolved		Protein	Total	Structure		Total	Mixed			Total
Nt	Anc	SNP	AA	Codon	AA	Codon	Residue	All	1	2	ST	1	2	3	MX
125	A	G	t	ACT	a	GCT	A23	4	1	1	2	1		1	2
146	A	G	t	ACT	a	GCT	A30	1		1	1				
153	C	T	s	TCC	f	TTC	A32	3	1	1	2		1		1
163	C	T	l	CTC	l	CTT	A35	1	1		1				
841	C	T	l	CTC	l	CTT	A261	1	1		1				
1204	C	T	g	GGC	g	GGT	A382	1	1		1				
1440	A	G	n	AAC	s	AGC	A461	1	1		1				
1440	A	G	q	CAA	q	CAG	B55								
1575	C	T	a	GCG	v	GTG	A506	2	1	1	2				
1575	C	T	s	AGC	s	AGT	B100								
1708	T	C	t	ACT	t	ACC	A550	1						1	1
1708	T	C	l	CTG	p	CCG	K24								
1786	C	T	l	CTC	f	TTC	C23	1		1	1				
1786	C	T	s	TCT	f	TTT	K50								
*1808*	*A*	*G*	*d*	*GAC*	*g*	*GGC*	*C30*	*1*	*1*		*1*				
*1808*	*A*	*G*	*x*	*TGA*	*w*	*TGG*	*K57*								
1866	C	T	f	TTC	f	TTT	C49	1	1		1				
1957	A	G	n	AAC	d	GAC	C80	1	1		1				
*1958*	*A*	*G*	*n*	*AAC*	*s*	*AGC*	*C80*	*2*		*1*	*1*	*1*			*1*
*1970*	*A*	*G*	*n*	*AAC*	*s*	*AGC*	*C84*	*2*	*1*	*1*	*2*				
2011	A	G	q	CAA	q	CAG	D12	1		1	1				
2098	T	C	r	CGT	r	CGC	D41	2	1		1			1	1
2119	C	T	r	CGC	r	CGT	D48	1	1		1				
*2149*	*C*	*T*	*c*	*TGC*	*c*	*TGT*	*D58*	*2*	*1*	*1*	*2*				
2173	C	T	v	GTC	v	GTT	D66	2	1	1	2				
2173	C	T	s	TCG	l	TTG	E7								
3281	T	C	s	TCC	p	CCC	F227	1				1			1
3594	A	G	k	AAA	r	AGA	F331	1					1		1
3734	A	G	k	AAA	e	GAA	F378	5	1	1	2	1	1	1	3
*3850*	*G*	*A*	*m*	*ATG*	*i*	*ATA*	*F416*	*1*	*1*		*1*				
4048	A	G	n	AAT	s	AGT	G10	2	1	1	2				
4202	C	T	r	CGC	r	CGT	G61	1		1	1				
4225	A	G	n	AAT	s	AGT	G69	4	1	1	2	1	1		2
*4530*	*A*	*G*	*t*	*ACC*	*a*	*GCC*	*G171*	*1*	*1*		*1*				
*4531*	*C*	*T*	*t*	*ACC*	*i*	*ATC*	*G171*	*2*		*1*	*1*			*1*	*1*
*4533*	*G*	*T*	*v*	*GTC*	*f*	*TTC*	*G172*	*2*		*1*	*1*			*1*	*1*
4741	A	G	t	ACT	a	GCT	H60	1	1		1				
4762	G	T	a	GCT	s	TCT	H67	1					1		1
4785	T	C	g	GGT	g	GGC	H74	1		1	1				
4786	A	G	i	ATC	v	GTC	H75	1	1		1				
4897	A	G	t	ACA	a	GCA	H112	1		1	1				
4967	G	A	g	GGT	d	GAT	H135	4	1	1	2	1	1		2
4988	G	A	g	GGT	d	GAT	H142	3	1	1	2			1	1
5025	T	C	l	CTT	l	CTC	H154	1	1		1				
5044	G	A	a	GCT	t	ACT	H161	2	1		1			1	1
5105	A	G	e	GAA	g	GGA	H181	1		1	1				
5110	A	G	t	ACT	a	GCT	H183	1		1	1				
5126	A	G	y	TAT	c	TGT	H188	1		1	1				
5127	T	C	y	TAT	y	TAC	H188	2	1	1	2				
							Totals	71	27	24	51	6	6	8	20

Note: Includes only substitutions detected in at least two squares on the same plate to reduce the likelihood of a call based on sequencing error. Genetic information for substitutions in overlapping genes is shown, but these substitutions are only counted once in the analyses. Rows in italics indicate substitutions that were seen in previous experiments with ID11 [26], [27].

st = structured transfer; mx = mixed transfer.

**Table 2:** Substitutions common to ST and MX environments.

	F378	H135	H142	*A23*	*A32*	*G69*	*G171*	C80-S	D41	G172	H161
ST-1	1	1	1	1	1	1	1		1		1
ST-2	1	1	1	1	1	1	1	1		1	
MX-1	1	1		1		1		1			
MX-2	1	1			1	1					
MX-3	1		1	1			1		1	1	1
Total	5	4	3	4	3	4	3	2	2	2	2

Note: A “1” indicates presence of the substitution anywhere on the plate. Amino acids in italics were seen in previous experiments with ID11 [26], [27]. In structured plates, excluding substitutions could occur on the same plate by occurring in different squares or being polymorphic in the same square.

We further quantified differences in genetic diversity between the ST and MX treatments by examining diversity measures at two scales: global (population or plate scale) and local (subpopulation or single-square scale). We estimated global diversity, 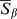, as the total number of unique mutational events per plate, averaged across within-treatment replicates. For structured-transfer plates, the global diversity was 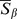 = 25.5±1.5 (mean ± SE) mutations per plate, while for mixed-transfer plates it was 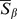 = 6.7±0.7. Mean global diversity was significantly lower in the MX treatment than in ST (t = 13.4, df = 3, p = 0.0009). This difference was also significant when we use the 10-square sub-sampling in the ST treatments that was used in MX (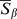 = 20.5±1.5; t = −3.13, df = 3, p = 0.002). Note that sampling only along the 10 squares in the “X” pattern results in an undersampling of ST diversity due to the patchiness of substitutions in ST plates (see Figure 1), whereas it is unlikely to affect the estimate of MX diversity since MX substitutions are spread across the entire plate.

We estimated local diversity, 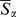, as the average number of mutations per square. The structured-transfer populations had 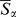
_ = _8.15±0.05 mutations per square and mixed-transfer populations had 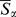
_ = _6.7±0.2. This difference was not significant (F_1,3_ = 2.96, p = 0.1836) when the square-wise variability per population is taken into account. To make these estimates comparable, only the counts from the squares that were sequenced in both the MX and the ST plates were used. There was a difference between the two treatments in the number of phage per square that were found at the end of the evolution experiment, with the ST plates having about 15-fold more phage than the MX plates (see Materials and Methods). However, there was no relationship between the number of mutations detected per square and the number of phage per square for each treatment. In the mixed-transfer populations the number of substitutions was the same for every square, therefore we looked for a correlation between number of phage and number of mutants across the three experiments (R = −0.03, t = 0.3394, df = 106, p-value = 0.6325). In the structured-transfer replicates there were different numbers of substitutions for each square, therefore we looked at the correlation for each replicate separately (ST-1: R = 0.21, t = 1.3, df = 34, p-value = 0.2023; ST-2: R = −0.17, t = −1.0005, df = 34, p-value = 0.3241). The fact that diversity estimates were not influenced by different, but very large, sample sizes is not surprising in view of the law of large numbers.

To ensure that differences in diversity were not due to differences in the numbers of phage being transferred in ST and MX treatments, we compared the number of phage transferred in the two sets of experiments. We estimated the average number of phage particles that were transferred from six different prongs on the replicate picker across all our treatments. In the ST treatment, 3.4×10^5^±7.4×10^4^ phage particles per prong were transferred. By comparison, in the MX treatment an average of 1.5×10^5^±2.6×10^4^ phage particles per prong were transferred. These minor differences in bottleneck sizes for ST and MX treatments are unlikely to have had a significant impact on diversity.

### Geographical Distribution of Mutations

We mapped both the spatial spread and frequency of each of the mutations by identifying where on the plate a given substitution was detected (Figure 1). Because the 454-based population sequencing does not provide much linkage information, we recorded each substitution separately. In Figure 1, we recorded the relative frequency in increments of 10% of a given substitution in each square using a gray scale, with white denoting absence and black fixation. These gray levels were determined from the fraction of 454 reads containing that substitution in the population sequencing of a given square.

An examination of Figure 1 indicates four important features of the data. First, the distribution of nucleotide substitutions was clustered in ST plates with a high level of variability in mutant frequency, owing to the slow spread of most mutants across the spatial landscape. In contrast, there was global spread and fixation of all substitutions in MX plates as anticipated from the experimental design. Second, in the structured treatment plates we observed squares where certain substitutions had reached fixation. As the distance from these squares increases, the frequency of that mutant declines, suggesting that squares with near fixation frequencies are the squares of origin for those substitutions. Third, eleven nucleotide substitutions (summarized in Table 2) appeared in both treatments, implying that these mutations may be beneficial for growth at high temperature and/or growth on a surface. Fourth, some substitutions that occur in the same gene appear to exclude each other: H135/H142, A23/A32, G69/G171. This pattern is seen most clearly in the MX plates where only one substitution of each pair is found on a plate (Table 2, Figure 1). The patterns for these substitutions in the ST plates are more subtle.

**Figure 3:**
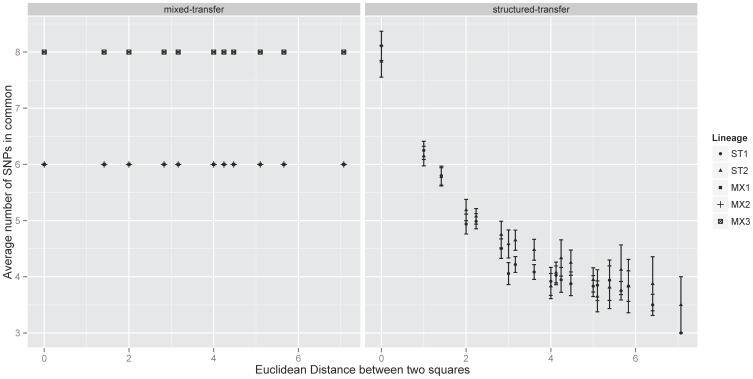
Mean number of substitutions shared by a pair of squares as distance between squares increases. For MX populations, each substitution was found across the entire plate, resulting in a lack of dependence on distance between squares when comparing shared substitutions. This suggests a single dominant lineage on each MX plate. In ST populations the patchy nature of substitutions is reflected in the decay in similarity with distance. This is consistent with multiple lineages dispersed over short distances on ST plates.

Restricted dispersal can create small patches of variability across a landscape, reducing the rate at which a mutation can spread and its relative abundance. This is illustrated by Figure 3 where the average number of shared mutations declines significantly with increasing distance between two squares in structured-transfer treatments (F_1,38_ = 138.6, r^2^ = 0.78, p = 3.03×10^−14^) but not in mixed-transfer treatments (F_1,31_ = 1.2×10^−27^, r^2^ = −0.03, p = 1). In each of the three mixed-transfer populations six to eight mutations occupied 100% of the plate after 550 generations, while only a single mutation fixed in the structured-transfer populations. This lowered fixation rate in structured-transfer populations translated to a reduced relative abundance. (The relative abundance of substitutions for a given treatment (ST or MX) was calculated as the average number of substitutions per square on a plate divided by the total number of substitutions on the plate, and averaged over the appropriate two (ST) or three (MX) replicates. It is a measure of substitution clustering on the plates.) On average a mutation in a ST population had a relative abundance of 0.37±0.03 across the plate, while in MX it was 1±0.0. Note that the value of 1 (with no variation) for relative abundance in MX is indicative of the global spread of all detected substitutions in that treatment.

Most of the changes that appeared in MX plates also appeared in ST plates. In particular, 5 of the 6 substitutions in MX-1 were also found in at least one of the ST replicates; similarly, 4 of 6 in MX-2 and 7 of 8 in MX-3 also arose in ST. Interestingly, those substitutions that are unique to the MX environment are restricted to only a single replicate. Furthermore, we sequenced 6 isolates from randomly chosen squares in both the ST and MX populations at the final time point (Table 3). All of the isolates from the MX treatment were identical, which is consistent with the notion that occasional mixing spreads beneficial mutants in a way that facilitates global selective sweeps. The ST isolates, by contrast, are quite different in the substitutions they contain. Additionally, in none of the isolates for either treatment do the “excluding” substitutions mentioned above appear on the same genome.

**Table 3:** Substitution locations in single isolates from six squares of ST-1 and MX-1 at generation 550.

Spatial Treatment	Grid #	Substitution locations								
structured	5F	–	A261/A*48	–	–	F378	G69	H75	H142	–
structured	4F	A23	A261/A*48	–	–	F378	G69	H75	H142	–
structured	2E	–	*–*	C30	–	F378	G69	–	H142	H161
structured	3D	–	*–*	–	–	F378	G10	–	H142	–
structured	4D	–	A261/A*48	–	F227	F378	G69	–	H135	–
structured	3B	–	*–*	–	–	F378	G69	–	–	H161
mixed	1B	A23	*–*	C80	F227	F378	G69	–	H135	–
mixed	3B	A23	*–*	C80	F227	F378	G69	–	H135	–
mixed	4D	A23	*–*	C80	F227	F378	G69	–	H135	–
mixed	6B	A23	*–*	C80	F227	F378	G69	–	H135	–
mixed	3C	A23	*–*	C80	F227	F378	G69	–	H135	–
mixed	3D	A23	*–*	C80	F227	F378	G69	–	H135	–

The site in italics is a silent substitution.

Note: Some columns contain multiple substitutions from the same gene to save space.

### Location of Mutations in the Viral Genome

In previous experimental work, ID11 was evolved at 37°C in liquid media and most of the first-step mutations were found in gene F, whereas second and third step mutations were scattered across the genome [26], [27]. It is likely that first step mutations were selected in the initial stages of adaptation to high temperature because they provided increased capsid stability [28]. Note that while this is not as high as the temperature used here, it is higher than the optimal growth temperature for ID11 of 33°C. The mutations that arose in our longer-term spatial evolution experiments were found in 10 of 11 genes in the ST populations and 7 of 11 genes in the MX populations. The broader scattering of mutations across the genome in our spatial experiments is undoubtedly influenced by the difference in time scale (550 generations of evolution versus tens of generations in [26], [27]) and the likelihood that the high-temperature stability problem was solved early on.

If mutations had accumulated randomly across the genome, then we would expect to see a relationship between the number of mutations observed in a gene and the length of that gene (Figure 2). To test whether the apparent differences are statistically significant, we conducted for each treatment a χ^2^ test comparing observed number of substitutions per gene vs. expected number based upon gene length. The same nucleotide substitution in two replicates was counted as two separate and independent mutational events. Furthermore, the ID11 genome has several overlapping genes, and genes B, E and K were excluded from this analysis. Unlike previous adaptations of ID11 in well-mixed environments, where gene F was the primary target of selection [26], [27], we found there was an over-representation of mutations in the structured-transfer treatments in genes G and H, while genes A and F were underrepresented (Figure 2; df = 6: χ^2^_ ST_^ = ^23.3 p<0.001; χ^2^_ MX_ = 3.9, p>0.79; χ^2^_ LQ_ = 34.2, p<0.0001).

### Fitness Assays on Individual Genotypes

**Figure 4:**
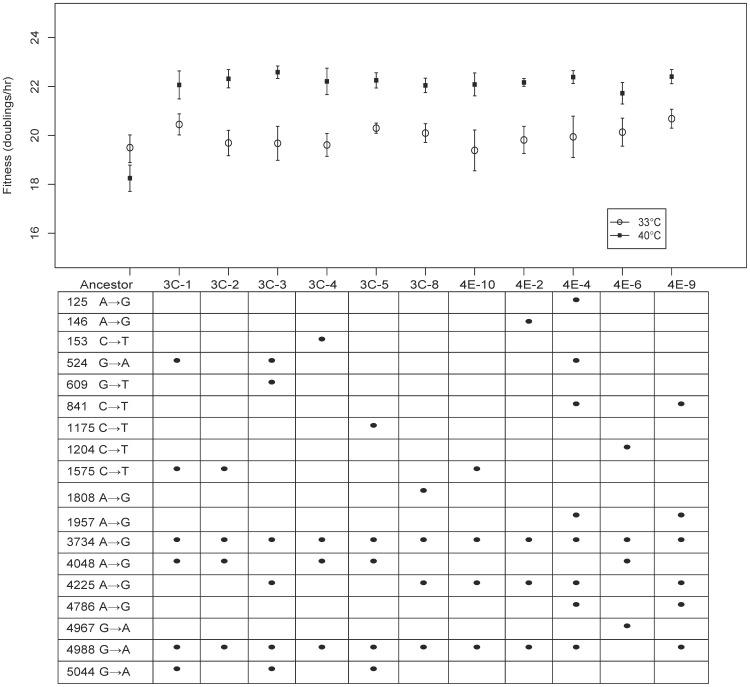
Plate fitness assays comparing ancestor to 11 isolates from the single evolved population ST-1. Mean and standard errors are based on 4 replicates per isolate growing at 40°C (squares) and 33°C (triangles). Nucleotide changes are indicated by dots in the table below each isolates results. Left column of table indicates nucleotide position followed by ancestral → mutant type. Right column indicates amino acid.

To determine if a subset of mutations detected in our experiments were beneficial at a phenotypic level, we compared the absolute fitness (number of doublings per hour) of 11 evolved genotypes to the ancestor at two assay temperatures: 33°C, the optimal temperature for the ancestor; and 40°C, the treatment temperature for the evolutions (Figure 4). Plaques were isolated at generation 550 from the ST-1 treatment. With the exception of the ancestor, all genotypes used in this fitness assay had one mutation in common: ^K^F378^E^ at nucleotide 3734. Each mutant isolate had 2 to 5 other substitutions. At 33°C the fitnesses of mutant isolates were not significantly different from the ancestor or each other (F_11,36_ = 0.49, p = 0.898). However, at 40°C the evolved isolates had significantly greater doublings per hour than the ancestor (F_11,36_ = 8.8, p = 3.6×10^−7^) but were indistinguishable from each other in this assay. This suggests that temperature, rather than host or other apects of laboratory conditions, is the major driver of adaptation in this system.

## Discussion

The goal of this study was to determine how spatial structure influences the fates of mutations that occur during adaptive evolution of viruses to thermal stress and to characterize the spatial distribution of the resulting genetic variation. We detected more global diversity (about three times as many substitutions plate-wide) in the structured-transfer treatments than in the mixed-transfer treatments. Local diversity (measured in smaller sub-regions), however, was very similar in both spatial treatments. The greater global diversity that arose when spatial structure was preserved was a manifestation of the patchy distribution of substitutions across the plate and fewer fixation events. This can be thought of as clonal interference in the first sense mentioned in the introduction. Populations with reduced spatial structure were characterized by fewer substitutions overall, but they were spread across all subpopulations. In other words, the occasional loss of spatial structure during mixed-transfer evolutions was enough to facilitate selective sweeps across the entire spatial landscape. Presumably, these sweeps resulted in clonal interference in the second sense mentioned in the introduction, with fitter mutants outcompeting some of the adaptive trajectories that flourished when spatial structure was maintained.

The theoretical predictions mentioned in the Introduction are based on spatial mathematical models that do not completely conform to our experimental system with its two types of periodic bottlenecks and phage (infecting bacterial hosts) instead of bacteria. Nevertheless, it is worth comparing theory and experiment to see which aspects of spatial evolution are strong enough to be robust to differences in some of the details. As expected, the evolved phage populations with preserved spatial structure led to significantly higher global diversity than in populations that were mixed at each transfer. The similarity in local diversity (measured in the 36 sub-squares) between spatial treatments does not correspond to any predictions from the available theory. This result is, no doubt, a function of the sizes of the subpopulations and would be interesting to explore further mathematically. The prediction of Habets et al. [11] that the initial distribution (localized or mixed) plays a role in the probability of fixation of a beneficial mutant certainly appears to have parallels in our experiments. Each incubation period that begins with a mixed transfer of phage corresponds to an initial distribution in which a given beneficial mutant is widely scattered across the spatial landscape. This is likely the main reason for the high probability of global fixation in our MX populations. Before a beneficial mutant has spread significantly in ST populations, it faces a challenge similar to the mutants that are localized initially in Habets et al. [11]. The prediction of parallel adaptation from the model of Ralph and Coop [4] is subtler to discern given the single time point observed in our study. This should become more transparent in our follow-up study that includes sequencing at multiple time points. The next paragraph provides some evidence for parallel evolution (the special case of parallel adaptation in which the same mutations arise in multiple locations).

We assert that a majority of the missense substitutions that occurred in both the mixed-transfer and structured-transfer plates at 40°C were adaptive. We provide four lines of evidence. First, we observed a high level of parallel evolution among the substitutions observed in our different replicate lines and with previous experiments on ID11; parallel evolution is a signature of adaptive substitutions in experimental evolution [29], [30]. Of the 40 different sites with amino acid substitutions seen in this experiment, 17 sites show parallel evolution within this experiment and seven are in common with previous experiments [26], [27] (Table 1). Because many of these substitutions are in common, this yields a total of 20 putatively adaptive changes. Additionally, within our ST plates, we found that there were likely multiple origins of some substitutions (i.e., multiple high-frequency foci on the same plate), which is another indication of parallel evolution. Secondly, we found an excess of non-synonymous changes over synonymous changes in both spatial treatments. Evidence that directional selection is acting on a related phage, φX174, exists if the ratio of missense-to-silent mutations is greater than 2.7 [29]. ID11 has the same genome structure, including the same overlapping reading frames that were taken into account in that calculation. Substitutions that are missense in one overlapping gene and silent in the other are counted as missense. Across all mixed-transfer replicates, only one silent substitution arose leading to a missense-to-silent ratio of 19 (19∶1), a strong indication of directional selection. Across all structured-transfer populations, the ratio of missense to silent changes was 2.6 (37∶14), which is a little less than the cutoff of 2.7. When the two spatial replicates are considered separately, ST-1 has a ratio of 2 (18∶9) and ST-2 has a ratio of 3.8 (19∶5), thus the large number of silent substitutions in ST-1 accounts for the ratio that is slightly lower than the cutoff. It is noteworthy that three of the silent substitutions in ST-1 arose in other populations, including the lone silent substitution in MX-3. Thus these silent sites may not represent neutral mutations. Alternatively, these could be neutral sites that are hitchhiking with other substitutions. Thirdly, we showed that the genomic distribution of changes was different than if mutations were randomly distributed in the genome, as might be expected for neutral mutations (Figure 2). Lastly we demonstrated that isolates from the ST-1 population were significantly more fit relative to the ancestor (Figure 4) at 40°C but not at 33°C. On average, fitness increased by 4 doublings per hour, or between a 5 and 10-fold increase in the population size per hour.

### Fixation, Clustering, Exclusion and Speed of Spread

Random mixing causes all mutant lineages to be in direct competition with each other, leading to sequential sweeps of the most-fit lineage [9], [31]. If this process swept to completion in our mixed-transfer populations, then isolates (single plaques) sampled from any square in a mixed-transfer plate should carry all the same mutations. Isolate sequencing (Table 3) from a single replicate mixed-transfer plate was consistent with the dominance of a single lineage. This would explain why average global diversity was equal to local level diversity in mixed-transfer populations (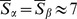). By contrast, mutations in the structured-transfer populations were localized and dispersal was restricted (Figures 1, 3) creating variation among different subpopulations in both the number and type of mutations found (Table 3). This led to a higher global diversity but reduced average local diversity: (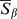 = 22, 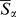 = 6.6).

There are several patterns to the distribution of substitutions in the structured-transfer populations that illustrate both patchiness and spread. The F378 substitution spread across all plates for both structured and mixed transfer. Several other substitutions reached very high frequencies across most of the plate, especially H142 and G69 in ST-1, and G69, H135 and A23 in ST-2. Other substitutions occurred less frequently in only small regions of the plates. Additionally, some substitutions occur in several different locations on a plate and are of note due to their fragmented distributions. We remark that the pattern of genetic variation across the plate was the same for the removed replicate (ST-3). Only the identity of substitutions revealed the possible switch with another structured passage experiment conducted at a lower temperature.

This range of different distributions across a plate suggests several interesting features of spatial structure that are not possible in a well-mixed environment. Due to the high density of phage across a plate at the end of each incubation period, we infer that there are multiple opportunities for a substitution to arise by mutation anywhere on a plate, likely in multiple genetic backgrounds. H60 on ST-1 and H181 on ST-2 illustrate this point; squares on the grid in which these mutations are found at low frequency are fairly well separated and are found with different substitutions. Adaptive substitutions can spread to neighboring squares at a rate determined by their fitness and their ability to compete with neighboring phage. A pattern of increasing to higher frequency (darker shading) and spreading to adjacent squares can be seen on the ST plates. Because of spatial structure, a particular phage genotype on a ST plate would not have found itself in competition with all the phage on the plate. This is unlike the situation on MX plates in which mutants first arise and spread locally and then are globally dispersed at the time of each mixed transfer, thus facilitating widespread competition. There is ample opportunity for local variants to increase to high numbers when structure is maintained. Eventually, locally adapted genotypes will spread into squares with other spreading genotypes, leading to direct competition between different genotypes that are well adapted to the high-temperature, plate environment. On the other hand, the F378 substitution appears to have such high fitness on plates at this temperature that it is fixed across all populations regardless of whether structure is maintained or not.

An interesting aspect of the spread of different genotypes can be seen for three pairs of substitutions in different genes. H135/H142, G69/G171, and A23/A32 are pairs of substitutions that do not appear to occur in the same phage or in the same location. One or the other is fixed on the mixed transfer plates and in the structured transfer plates the distributions of these pairs is complementary (cf. H135/H142 and G69/G171 on ST-2, A23/A32 on ST-1). These substitutions coexist on the plate, but are generally found in different squares or in opposing frequencies in the same square (Figure 1). Sequenced isolates also never have both of these paired substitutions (Table 3; Figure 4). The same pattern was seen for the H135/H142 pair during replicate laboratory adaptation of three other G4-like phage taken from the wild [32]. This pattern suggests that these ‘excluding’ pairs may have the same adaptive function in the phage in which they occur, such that once one mutation of a pair occurs the second is no longer adaptive.

Of course, patterns of patchiness and restricted spread may also be the remnants of substitutions that are losing to more fit genotypes rather than those that are starting to sweep through the population. Future studies will provide temporal information about the spread of these substitutions over the length of these experiments.

### Relationship to Phage Biology

ID11 has been used in previous evolution experiments in which phage were grown on *E. coli* C at 37°C in batch cultures at low density [26], [27]. Some substitutions that arose in our experiments also arose in these other experiments (Table 1, see italics), suggesting that these substitutions are not specific adaptations for growth on plates. Most of these parallel or convergent substitutions were found in proteins C, D and G with only a single mutation arising in F. Comparing the sequence of our ancestral strain of ID11 to other wild, G4-like phage, we find that the substitution that swept through all plates in both experiments ^A^3734^G^ is a G in all but one other phage, and this substitution is therefore a reversion to the dominant wild genotype and amino acid [33]. Additionally, ^C^4531^T^, which changes G171 to an isoleucine, is found in two of the wild phages. This pattern of reversion has also been seen for high frequency changes during experimental evolution with the closely related phage, **φ**X174 [30].

Of the 11 mutations found in common between structured- and mixed-transfer populations, three were in the major spike protein (^N^G69^S^, ^T^G171^I^, ^V^G172^F^) and three were in the pilot protein (^G^H135^D^, ^G^H142^D^, and ^A^H161^T^). G4-like viruses encase their single-stranded circular DNA in a capsid, which has twelve vertices [34]. Each of these 12 vertices are formed by pentamers of F proteins, and on these vertices are spikes consisting of five G proteins. The common substitutions in protein G affect amino acids that lie on the interface between the G pentamers and the underlying F pentamers, and thus may affect capsid stability. In total, there were eight nucleotide substitutions in the gene for protein G. The two substitutions in H that appear to be mutually exclusive, ^G^H135^D^ and ^G^H142^D^, are at the start of the predicted coiled coil domain, and the third, ^A^H161^T^, is within the predicted coiled coil domain [35].

Two other mutations that were common to both the structured- and mixed-transfer populations were ^T^A23^A^ and ^S^A32^F^. Gene *A* encodes the replication initiation protein A, and includes sequence that encodes proteins A*, B and part of K [34]. Protein A* is the C-terminal 2/3 of protein A and has the ability to nick DNA at specific sequences; its biological function is unknown. Protein B is encoded in an overlapping reading frame of *A* and is the internal scaffolding protein. Protein K is encoded by an overlapping reading frame covering both genes *A* and *C*, and its function is unknown. Another seven substitutions are found in gene *A*, four of which were missense in at least one of these genes. Previous evolution experiments with G4-like phage have not produced substitutions in gene *A* [26], [27], [32]. These previous experiments were specifically designed to minimize intracellular competition among phage and had significantly fewer phage generations. The functions of the amino acids in A at 23 and 32 are unknown; therefore, understanding their significance awaits further research.

## Materials and Methods

### Strain and Culture Conditions

The bacteriophage ID11 (GenBank Accession: AY751298) was originally isolated from the barnyards at the University of Idaho [33]. It is a single-stranded DNA virus in the G4-like clade of the family Microviridae [26]. We used this bacteriophage because the genome is small (5577 bp), all 11 of its genes are annotated, and it has not been adapted to laboratory conditions. Each of the three replicate lines for the two treatments was started from a different isolate of the ID11 ancestral strain grown at 37°C (e.g. ST-1 and MX-1 came from the same isolate as did ST-2 and MX-2, and ST-3 and MX-3.). All three ancestral plaques carried a single amino acid replacement in protein D, ^N^D131^S^, (^A^2367^G^) when compared to AY751298.

We used the standard lab host *Escherichia coli* C [36]. All experiments were carried out on square plates (3.5 in × 3.5 in × 0.5 in) with a 6×6 array of squares (½×½ in) etched on the bottom of each plate (see Figure 1). The base of each plate was LB agar (10 g/L NaCl, 10 g/L Tryptone, 5 g/L yeast extract, 15 g/L agar), which was then layered with host cells in 4.5 mL of soft top agar (as above except with 7 g/L agar and supplemented with 200 µL of 2 mM CaCl_2_).

To create fresh plates of naïve host cells for each incubation period, *E. coli* C from a previously prepared and frozen stock was grown for one hour at 37°C in 10 mL of LB broth until log-phase growth was reached (∼1×10^8^ cfu/mL). In order to ensure that the starting density of hosts did not vary in magnitude, we checked that the OD readings after 1 h of *E. coli* C log phase growth were within 2 standard deviations of the mean before adding cells to the top agar. Finally, to prevent the persistence of resistant host cells, we killed all hosts at the end of each incubation period by using parafilm to seal inverted agar plates to plates containing a chloroform-soaked filter and incubating at room temperature for one hour.

### Evolution Experiment

Our evolution experiment was carried out at 40°C and had two treatments corresponding to different levels of spatial structure: structured-transfer and mixed-transfer, defined above. For each of the two treatments, three replicate populations were evolved by transferring viral populations to fresh plates with naive *E. coli* hosts and then incubated at 40°C for 4 h, which corresponds to ∼11 *E. coli* population doublings. We limited incubation times to prevent increasing the frequency of resistant host cells and complicating the selective environment [37].

After incubation and killing of resistant cells, the “structured-transfer” (ST) populations were transferred from one plate to another using a 384-prong replicate picker. Because physical orientation of the plate was important, we maintained a consistent transfer pattern. In the “mixed-transfer” (MX) treatments, viral populations were scraped from the plates into cell culture tubes filled with 2 mL of LB and 200 µL of chloroform and vortexed. The complete supernatant was then plated out in top agar and allowed to harden slightly. After randomly mixing the phage subpopulations in a liquid environment, a sample of the viral subpopulations were transferred from this pre-transfer plate onto a fresh plate with naïve hosts using the 384-prong picker. A replicate picker was also used in the mixed-transfer plates to ensure, as in the structured-transfer plates, that only subsamples of the phage populations were transferred. For both ST and MX treatments, a single passage constituted the transfer of phage onto new hosts and incubation at 40°C for 4 h. This was enough time for a phage plaque to spread across multiple picker prongs, ensuring an intermingling of the subpopulations. The evolution experiments continued for 50 passages, which corresponds to approximately 550 viral generations.

The structured- and mixed-transfer experiments were performed sequentially to prevent contamination between the two levels of structure. To avoid cross-contamination between replicate plates within each experiment, the replicate picker was heated in boiling water for 2–5 minutes and then cooled in a 100% ethanol bath. Furthermore, we ran a set of empty control plates (top agar and *E. coli* C) stuck with the sterilized replicate picker between replicates. In the event that phage plaques were detected on our control plates, we restarted the experiment by going back two passages.

Structured-transfer plates were archived after every second passage by scraping each of the 36 squares into different tubes using a sterilized metal spatula. Because transfer of the mixed transfer plate disrupts spatial structure, mixed-transfer plates were archived after every 10^th^ passage by making replicate plates for these passages, with one plate archived as above and the other used for transfer to fresh hosts. For long-term storage of phage samples, LB broth and 25% w/v glycerol was used. To prevent cross-contamination between squares, the spatula was flamed until red hot and then cooled in an ethanol wash and air-dried. Further, periodically the spatula was tested for contaminant phage by lightly dragging it across an empty plate of top agar and *E. coli C* to see if any phage plaques formed. These tests were always negative.

### Sequencing

Each plate from the structured-transfer experiments was treated as a population consisting of 36 subpopulations (6×6 grid of squares). Each of the 36 subpopulations under the structured-transfer treatment and 10 subpopulations (see Figure 1) from the mixed-transfer treatment were sequenced from the 50^th^ passage (550^th^ generation) on a Roche 454 Genome Sequencing FLX (information available from the authors upon request). DNA was purified from each subpopulation, and then amplified using 24 individually-tagged primer pairs (Table 4) on a Fluidigm Access Array System. The tags associated each sequence with its subpopulation. The amplified products were then combined and run on a quarter plate of the 454 FLX. On average the coverage was 50x or greater for each sample (i.e., subpopulation). On average, the concentration of phage in ST squares (across all squares and all replicates) was 1.66×10^9^ per mL (±1.56×10^8^), while the concentration in MX squares was 1.14×10^8^ per mL (±1.72×10^7^). Based on the fact that subpopulations were sequenced at 50x coverage, these differences in concentration are not significant.

**Table 4:** Oligonucleotides used to amplify and sequence structured-transfer and mixed-transfer populations.

Primer Name	Primer Sequence (5′ to 3′)
UG49A	AATATGCCTCCCATCAAACGG
UG2702A	AGAGTCACCAGCAACAACAGG
UG2495B	TAATGAAAAAATCAATTCGCC
UG225B	TCACGGCGAATACCATTTG
UG4067	GAGTCGAACGACGATGATTTTGGG
UG49A	AATATGCCTCCCATCAAACGG
UG548A	AAACCCCTCGGTAATGACC
UG754AR	GGCAGTACGCATAGCATTC
UG937A	TGCTATCAGTATTTTTGTGTG
UG1447A	ACCCCGTTCAACAACATCTTG
UG1997A	TGTCTAAATCAAACGAATCTG
UG2316A	CGTCAATGGTGTTGAACGCCC
UG2495B	TAATGAAAAAATCAATTCGCC
UG3162B	TGAAAACATGACTACTGGTAC
UG3645B	AATTCAAAATCGCTGAGGG
UG4050B	TTCATTTCTAAGCACAATG
UG4501B	CTACTATCTCTGGCGTCCT
UG4947B	ACCCATGGGAACGTGCTGG
UG5435B	GCTAAGGACGTGTCCAATG

Additionally, 12 isolates from 6 diagonal squares in ST-1 and from MX-1 were characterized using Sanger sequencing on an ABI 3730 using Big Dye sequencing reagents as previously described [29].

### Fitness Assays

We isolated eleven plaques from the final time point of the ST-1 structured-transfer replicate; six of the plaques were from square 3C and five were from square 4E. The genotypes of all 11 evolved isolates were characterized using Sanger sequencing. Absolute fitness, the number of phage doublings per hour on plates, was assayed for each evolved genotype and the wild type ancestor at two temperatures, 33°C and 40°C. Each plaque was sampled from frozen stocks and then titered to determine the volume required for initiating the assay. *E. coli* C was grown with aeration to ∼10^8^ cells/mL at 37°C in 10 mL of LB broth. Then 120 phage particles (*N_0_*) were plated with 200 µL of these host cells in 3.5 mL of top agar and incubated at the assay temperature. After one hour, growth was terminated by scraping the top agar from each plate into cell culture tubes containing 1 mL LB broth and 115 µL chloroform. This was then centrifuged for 20 minutes at 4°C. The resulting supernatant was titered and incubated at the assay temperature. Plaques were counted to obtain our final population size of phage (*N_1_*). Fitness was calculated as *W* = log_2_(*N_1_*/*N_0_*)/1 (doublings per hour).
